# Redox Balance-DDR-miRNA Triangle: Relevance in Genome Stability and Stress Responses in Plants

**DOI:** 10.3389/fpls.2019.00989

**Published:** 2019-08-02

**Authors:** Sara Cimini, Carla Gualtieri, Anca Macovei, Alma Balestrazzi, Laura De Gara, Vittoria Locato

**Affiliations:** ^1^Unit of Food Science and Human Nutrition, Campus Bio-Medico University of Rome, Rome, Italy; ^2^Department of Biology and Biotechnology “L. Spallanzani”, University of Pavia, Pavia, Italy

**Keywords:** redox balance, DDR, miRNA, redox-sensitive TFs, cell cycle checkpoints

## Abstract

Plants are continuously faced with complex environmental conditions which can affect the oxidative metabolism and photosynthetic efficiency, thus leading to the over-production of reactive oxygen species (ROS). Over a certain threshold, ROS can damage DNA. DNA damage, unless repaired, can affect genome stability, thus interfering with cell survival and severely reducing crop productivity. A complex network of pathways involved in DNA damage response (DDR) needs to be activated in order to maintain genome integrity. The expression of specific genes belonging to these pathways can be used as indicators of oxidative DNA damage and effective DNA repair in plants subjected to stress conditions. Managing ROS levels by modulating their production and scavenging systems shifts the role of these compounds from toxic molecules to key messengers involved in plant tolerance acquisition. Oxidative and anti-oxidative signals normally move among the different cell compartments, including the nucleus, cytosol, and organelles. Nuclei are dynamically equipped with different redox systems, such as glutathione (GSH), thiol reductases, and redox regulated transcription factors (TFs). The nuclear redox network participates in the regulation of the DNA metabolism, in terms of transcriptional events, replication, and repair mechanisms. This mainly occurs through redox-dependent regulatory mechanisms comprising redox buffering and post-translational modifications, such as the thiol-disulphide switch, glutathionylation, and S-nitrosylation. The regulatory role of microRNAs (miRNAs) is also emerging for the maintenance of genome stability and the modulation of antioxidative machinery under adverse environmental conditions. In fact, redox systems and DDR pathways can be controlled at a post-transcriptional level by miRNAs. This review reports on the interconnections between the DDR pathways and redox balancing systems. It presents a new dynamic picture by taking into account the shared regulatory mechanism mediated by miRNAs in plant defense responses to stress.

## Introduction

The maintenance of the cellular redox balance is a major biological attribute influencing growth, development and survival in plant and animal systems (de Pinto et al., 1999, 2015; Pellny et al., 2009; Chiu and Dawes, 2012). In animal systems, a mild oxidative environment has been observed to activate a signaling pathway leading to cell proliferation (Menon et al., 2003; Menon and Goswami, 2007). Interaction between the epidermal growth factor and their specific receptor stimulates cell proliferation by the generation of a low amount of reactive oxygen species (ROS; Menon and Goswami, 2007). In plants, a strong correlation between the cellular redox state and cell cycle block has been clearly observed in the root quiescent center, a group of spatially defined cells that are blocked in G0 (Jiang and Feldman, 2005; Jiang et al., 2006; Dinneny et al., 2008). An increase in ROS production generally causes a cell cycle arrest before the activation of the cell death program (Chiu and Dawes, 2012; de Pinto et al., 2012). As a common feature of eukaryotic organisms, it has been hypothesized that cell cycle progression is driven by an intrinsic redox cycle consisting in regulated reductive and oxidative phases (Chiu and Dawes, 2012). Glutathione (GSH), the most abundant non-protein thiol in the cell, seems to be a major actor in the redox fluctuations normally occurring during cell proliferation in animal and plant cells (García-Giménez et al., 2013). Alterations in the cell redox potential may also be responsible for the abnormal proliferation of cancer cells which have a “constitutive” decrease in the cellular redox potential, and therapies able to adjust their cellular redox balance have been proposed (Hoffman et al., 2001, 2008). In plants, phythogen toxins blocking cell proliferation induces an alteration in GSH fluxes between nucleous and cytosol (Locato et al., 2015). Thus, sensing the redox state at tissue, cellular and subcellular levels is needed to accurately allow cell cycle progression in the right redox environmental conditions, linking the cell stress response to the cell cycle checkpoint pathway (Pearce and Humphrey, 2001).

The maintenance of the cellular redox balance is also a crucial attribute influencing plant development. Plant embryogenesis has indeed been correlated to a shift in the cell environment toward a more oxidized state (Belmonte and Stasolla, 2007; Stasolla et al., 2008; Becker et al., 2014) and the circadian clock also seems to be regulated at the redox level and vice versa (Lai et al., 2012). Moreover, the cell redox state is intrinsically correlated to the cell metabolic status and consequently it is presumed to be tightly linked to cell energy efficiency. In aerobic organisms, perturbations in the cell redox status are reflected in metabolic efficiency, calculated as the ratio between oxygen consumption and ATP production (Giancaspero et al., 2009). In line with this, in plants, environmental stressing conditions that perturb the cellular redox status have been found to impair the mitochondrial metabolism (Vacca et al., 2004; Valenti et al., 2007). Thus, metabolic efficiency can be monitored by assaying the mitochondrial respiration pathway. In the yeast model, the metabolic cycle, which consists of respiratory (oxidative phase) and fermentative/non-respiratory (reductive phase) phases, seems to be synchronized to cell cycle progression, with mitosis and DNA replication occurring during the reductive phase and G1 during the oxidative phase (Tu et al., 2005). This synchronization may act as a protective mechanism toward genome integrity, thus enabling DNA synthesis to occur in a non-oxidative environment (Chen et al., 2007).

**FIGURE 1:**
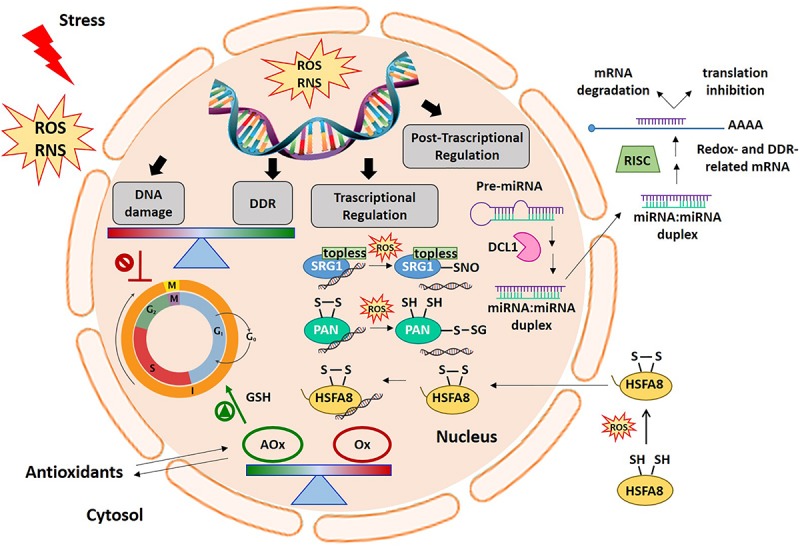
Cellular redox balance-DDR-miRNA triangle. An increase in ROS production generally occurs early under different stress conditions. ROS and redox signals move through different cell compartments. In the nucleus, ROS accumulation can cause DNA damage thus inducing cell cycle arrest. The DDR specifically aims to help plants cope with the negative effects of genotoxic stress. Alterations in antioxidant and oxidant balance in the nucleus are required to promote cell cycle progression in the right redox environment. In this context, ROS and redox signals are involved in the regulation of gene expression at transcriptional and post-transcriptional levels. The picture shows some examples of the redox dependent transcriptional mechanisms involving some redox-sensitive TFs, such as SRG1, PAN, and HSFA8, in gene expression regulation. At the post-transcriptional level, the figure also shows the modulation of redox- and DDR-related target mRNAs by miRNAs. AOx, antioxidants; DCL1, DICER-like1; DDR, DNA damage responses; HSFA8, heat shock factor A8; miRNA, microRNA; Ox, oxidants; PAN, PERIANTHIA; RNS, reactive nitrogen species; ROS, reactive oxygen species; SRG1, SNO-regulated gene 1.

Plant exposure to stressful conditions, both exogenous (solar UV radiation, high soil salinity, drought, chilling injury, air and soil pollutants including heavy metals) and endogenous (metabolic by-products) in nature, can compromise genome integrity. Due to their sessile lifestyle, and the presence, for all the lifespan, of a small population of the same meristematic cells continuously dividing for allowing organism growth, plants have evolved various strategies to cope with environmental constraint conditions (Spampinato, 2017). Among these, the continuous exposure to sunlight represents a dramatic challenge to genome integrity and to genome transmission to the subsequent generation (Roy, 2014). The DDR specifically aims to aid plants to cope with the detrimental effects of genotoxic stress. DDR is a complex signal transduction pathway, which detects DNA damage signals and transduces those signals to execute cellular responses. Both redox systems and DDR pathways are usually tightly regulated through the coordinated activities of cellular oxidants/antioxidants and DNA damage/repair-signaling pathways (Figure 1). It is well-known that intracellular ROS acts both as a cellular damaging compound and as a signaling molecule, all depending on its concentration and localization (Foyer and Noctor, 2003; Jeevan Kumar et al., 2015; Mittler, 2017). Links between ROS and DDR pathways have been hypothesized but not yet clearly demonstrated. For instance, studies on animal cells treated with neocarzinostatin (a radiomimetic that causes the formation of double-strand breaks) have shown that ROS induction is partly mediated by increasing levels of histone H2AX, a biomarker for DDR (Kang et al., 2012). Hence, ROS generate DNA damage while being regulated by the DNA damage-signaling pathways.

All the evidence outlined above makes controlling the cell redox balance a major regulator of virtually all plant metabolic re-arrangements occurring in growth, development and defense strategies. Regulation of all the metabolic transitions experienced by DNA (first and foremost, transcription, replication, and repair) within the cells is expected to be tightly connected with redox signaling pathways. Furthermore, the maintenance of cellular redox homeostasis and genomic integrity can be modulated by the activity of microRNAs (Figure 1; miRNAs). This review reports on the influence of various redox active systems on DNA damage response pathways and plant transcriptome as well as on post-transcriptional gene expression regulation mediated by miRNA.

## Ddr as a Keeper of Genome Stability

DNA damage response is an evolutionary conserved, complex network that includes signal transduction pathways composed of sensors, transducers, mediators, and effectors, dedicated to safeguard genome integrity. Several comparative studies have highlighted the conserved features of the core DDR machinery across eukaryotes, including plants and mammals, as well as the presence of unique characteristics in plants (DiRuggiero et al., 1999; Singh et al., 2010; Spampinato, 2017; Nikitaki et al., 2018). Most DDR components are ancestral genes that appeared early in the phylogenetic tree and subsequently expanded and shaped throughout evolution. Based on the detection of a DNA lesion by dedicated sensors, various pathways may be triggered, leading to the activation of cell cycle checkpoints, DNA repair, or programmed cell death (PCD). Endoreduplication, consisting of DNA replication in the absence of cytokinesis, represents a plant-distinctive process, which is also part of DDR (Yoshiyama et al., 2013b).

Most of the knowledge regarding DDR and DNA repair pathways, gained through decades of studies on yeast, bacteria, and mammals, has highlighted its function in plant biology (Spampinato, 2017). Indeed, functional and/or structural homologs of various DDR factors found in animals have been identified in model plants such as Arabidopsis (Spampinato, 2017) and *Medicago truncatula* (Balestrazzi et al., 2011). Some exhaustive examples are: MRE11 (Meiotic Recombination 11), RAD51, NBS1 (Nijmegen breakage syndrome 1) proteins, constituting the MRN complex, and RPA (Replication protein A). The MRN complex is required for double-strand break (DSB) recognition in the DDR pathway involving ATM (Ataxia Telangiectasia Mutated) kinase (Yoshiyama et al., 2013a), while RPA binds to single-stranded DNA (ssDNA) lesions associated with DNA replication in a pathway involving the ATR (ATM and Rad3-related) protein. The ATM and ATR transducers amplify and transduce signals to subsequent effectors through a phosphorylation-mediated cascade of events resulting in the activation of downstream processes (cell cycle arrest with the critical choice between DNA repair and PCD) (Culligan et al., 2004, 2006; Yoshiyama et al., 2013a, b). For instance, ATM and ATR transducers induce phosphorylation of the histone-variant H2AX (Dickey et al., 2009, Yuan et al., 2010) which, in the γH2AX phosphorylated form, acts as a DNA damage signal and recruits several proteins to the DSB site (Petrini and Stracker, 2003; Yoshiyama et al., 2013a). In yeast and mammals, after ATR activation, serine/threonine-protein kinases CHK1 (checkpoint kinase) and CHK2 were phosphorylated by ATR and ATM, respectively, with a consequent activation of cell-cycle checkpoints (Bartek et al., 2001; Chen and Sanchez, 2004). Arabidopsis appears to have no CHK1 and CHK2 orthologs. Considering that some of the substrates of CHK1 and CHK2 in animals, such as the mediator BRCA1 (breast cancer susceptibility gene 1), and E2F1 (E2F Transcription Factor 1), are also present in plants (Lafarge and Montané, 2003; Inze and de Veylder, 2006), it has been suggested that other kinases may work as functional homologs of CHK1 and CHK2 (Yoshiyama et al., 2013b). Studies on the Arabidopsis *atm* and *atr* mutants have shown that in addition to the conserved function in DDR, ATM and ATR play a different role in the life of plants (Garcia et al., 2003; Culligan et al., 2004). Yan et al. (2013) reported an intriguing finding linking the plant immune system to DNA damage. They demonstrated that the plant hormone SA induces DNA damage in the absence of a genotoxic agent, and the DDR components, ATR and RAD17 (radiation sensitive) are required for adequate plant immune responses, thus suggesting the role of DDR in the defense against pathogens. In contrast, Rodriguez et al. (2018) reported DNA damage as a consequence of autoimmune response rather than actively produced host-DNA damage aimed at stimulating resistance to pathogens.

The various factors involved in DDR are temporally and spatially regulated and activated through the action of mediators that recruit additional substrates and control their association with damaged DNA (Stewart et al., 2003; Stracker et al., 2009). Several mediators are known in human cells, such as MDC1 (mediator of DNA-damage checkpoint protein 1), 53BP1 (p53-binding protein), BRCA1 (Breast cancer susceptibility 1), related to the ATM pathway, TOPBP1 (topoisomerase 2-binding protein 1), and CLSPN (Claspin), involved in the co-regulation of the ATR pathway. Plants lack counterparts for some DDR mediators (e.g., MDC1 and 53BP1) (Yoshiyama et al., 2013b; Nikitaki et al., 2018). However, there are DDR components exclusively found in plants, such as SMR (Siamese-related) cyclin-dependent protein kinase inhibitors, some chromatin remodelers (CHR complexes), and several DNA and histone methyltransferases such as CMT3 [DNA (cytosine-5)-methyltransferase 3], SDG26 (SET domain group 26), SUVH5 (histone-lysine N-methyltransferase, H3 lysine-9 specific) (Nikitaki et al., 2018). Interestingly, the p53 effector, which is a TF acting as tumor suppressor in animal cells, does not exist in plants. In animals, the master regulator p53 rules the fate of the cell following DNA damage, which triggers cell-cycle arrest and then DNA repair or apoptosis (Helton and Chen, 2007). A similar role in plants has been ascribed to the TF SOG1 (suppressor of gamma response 1), a component of the NAC (NAM-ATAF1/2-CUC2) family (Preuss and Britt, 2003; Yoshiyama et al., 2009). SOG1 regulates more than 100 genes and similarly to p53, induces several pathways including cell cycle arrest, DNA repair, PCD, and endoreduplication (Yoshiyama et al., 2013b; Yoshiyama, 2015). It is thus clear that most of the DDR factors are well preserved in animals and plants, although various key components are unique to plants.

### DNA Damage Repair Mechanisms Activated by DDR Pathways

Of the pathways triggered by DDR effectors, DNA repair mechanisms are crucial in maintaining genome integrity. Several pathways are involved in the correction of various types of DNA lesions including: (1) direct repair (DR) or photoreactivation (Jiang et al., 1997), (2) mismatch repair (MMR), (3) base- and nucleotide excision repair (BER, NER) (Shuck et al., 2008; Peña-Diaz and Jiricny, 2012; Jiricny, 2013), (4) double-strand break repair (DSBR), which includes non-homologous end joining (NHEJ), and homologous recombination (HR) mechanisms (Puchta and Hohn, 1996).

The DR is a light-dependent pathway that relies on the activity of flavoenzymes, called photolyases, carrying the two electron-reduced forms of FAD (FADH) as photocatalysts (Sancar, 2003). After binding to the DNA lesion, the enzymes remove the damage following absorption of blue light in the 300–600 nm range (Tuteja et al., 2009). The activity of photolyases is specific to plants, since it seems to be absent in humans and other placental animals (Essen and Klar, 2006). On the other side, MMR is present in all organisms, and corrects replication and genetic recombination errors, which result in poorly matched nucleotides. In eukaryotes, the lesion detected by MutS homolog (MSH) proteins is repaired through enzymatic complexes operating an endonucleolytic cut on the neo-synthetized strand, thus restoring the correct sequence through the action of specific DNA polymerases (Marti et al., 2002; Spampinato, 2017).

The BER mechanism is responsible for the repair of damaged single bases resulting from deamination, alkylation, oxidized bases, abasic (apurinic and/or apyrimidinic, AP) sites, and single-strand breaks (SSBs) (Tuteja et al., 2009). It consists of the excision of the damaged base by a DNA glycosylase followed by the consecutive action of at least three enzymes, an AP endonuclease, a DNA polymerase, and a DNA ligase (Stivers and Jiang, 2003). The 8-oxoguanine DNA glycosylase (OGG1), uracil DNA glycosylase (UNG), and formamidopyrimidine DNA glycosylase (FPG), are some examples of plant DNA glycosylases with roles in stress responses (Gutman and Niyogi, 2009; Macovei et al., 2011). Aside being extensively studied in model plants, the pathway has also been characterized in potato mitochondria where it is mostly involved in the repair of DNA damage related to ROS production (Ferrando et al., 2018). While BER removes small DNA lesions, the NER pathway repairs the main DNA lesions causing extensive distortion in the double helix, such as UV-products and bulky covalent adducts (Kunz et al., 2005). The NER mechanism has mostly been studied in Arabidopsis and rice and investigations on NER genes have also been conducted in other plants such as poplar and sorghum (Singh et al., 2010; Spampinato, 2017). Proteins belonging to the RAD family are involved in DNA lesion recognition in NER. For instance, the RAD23 family of genes has been well characterized in Arabidopsis by developing multiple mutant plants. The triple and quadruple mutants for *rad23a*, *rad23b*, *rad23c*, and *rad23d* genes have shown clear phenotypic changes resulting in dwarfed plants or reproductive lethally mutants. However, single and double mutants have not shown evident differences, thus suggesting a mostly overlapping function of the four genes.

Repairing the DSBs is mostly carried out by DBSR systems. Studies on DBSR mechanisms have been increasing, not only because of their importance in DNA repair but also as tools to modify plant genomes (Baltes and Voytas, 2015; Sprink et al., 2015). DBSR mechanisms mainly include the HR and NHEJ pathways. HR occurs only when two DNA duplexes contain extensive homology regions, while NHEJ enables DSBs to be repaired in the absence of sequence homology. Given the requirement of a sister chromatid as a template, HR is restricted to the S and G_2_ phases of the cell cycle, while NHEJ is active throughout the cell cycle and does not rely on a template (Brandsma and Gent, 2012). The error-free HR pathway uses several enzymes including the ssDNA-binding protein RAD51 recombinase (Chapman et al., 2012; Spampinato, 2017). The balance between the HR and NHEJ pathways is essential for genome stability. Besides the well−characterized Ku−dependent NHEJ pathway (classical non-homologous end-joining, C-NHEJ), an XRCC1(X-ray cross-complementation group)-dependent pathway (alternative non-homologous end-joining, A-NHEJ) has been observed both in humans and in plants (Decottignies, 2013; Bétermier et al., 2014; Williams et al., 2014; Sfeir and Symington, 2015; Spampinato, 2017). C-NHEJ is dominant in the G_1_ and G_2_ phases of the cell cycle, while A-NHEJ preferentially acts in the S-phase (Karanam et al., 2012; Truong et al., 2013). A-NHEJ takes place in the absence of key C-NHEJ factors, and requires the alignment of microhomologous sequences. The pathway is thus also referred to as microhomology-mediated end-joining (MMEJ). Unlike HR, the lack of a homology sequence in NHEJ leads to an error-prone type of repair, frequently resulting in small insertions, deletions, or substitutions at the break site (Chapman et al., 2012).

### DDR in Relation to Redox-Based Mechanisms

Redox-based mechanisms would seem to play a key role in the modulation of DNA damage sensing, signaling, and repair. Although there is extensive knowledge in animal systems (Kim et al., 2013; Mikhed et al., 2015; Somyajit et al., 2017), there are few reports on redox signaling and redox-mediated control of DNA repair in plants (Zhang, 2015). Due to the complexity of such molecular networks and in an attempt to draw a representative picture of the state of the art in the plant kingdom, attention has focused on specific players that have been identified at the crossroads of the redox and DDR pathways.

One case relates to Fe-containing proteins (e.g., Fe–S cluster proteins and hemoproteins) which use Fe as a cofactor and play critical roles in several aspects of genome maintenance, including telomere maintenance and cell cycle control in both animals and plants (Zhang, 2014, 2015). In Arabidopsis, several Fe-containing proteins with key functions in genome stability, including DNA helicases and DNA glycosylases, have been characterized. For example, RAD3 (also known as UVH6), the plant homolog of the human XPD and yeast RAD3 proteins, is an essential helicase required for NER function (Liu et al., 2003). Among the known 26 DNA glycosylases, only DEM (DNA glycosylase DEMETER), DML1 (DEMETER-like 1, AtROS1), DML2, and DML3 proteins contain a Fe-S cluster and participate in DNA methylation (Ortega-Galisteo et al., 2008). The biogenesis of Fe-S proteins requires dedicated cluster assembly pathways (Lill and Mühlenhoff, 2008). The highly conserved cytosolic Fe-S cluster assembly (CIA) machinery is required for the transfer of these clusters to target proteins, including those involved in genome maintenance, and impairment of the CIA pathway possibly compromises genome integrity (Netz et al., 2014). Other pathways are located within subcellular compartments such as ISC (iron–sulfur cluster) in mitochondria and SUF (sulfur mobilization) in plastids (Couturier et al., 2013). Mutations that target genes coding for the CIA subunit, including AE7 (AS1/2 enhancer 7) and ATM3 (ABC transporter of the mitochondrion 3), result in DNA damage accumulation and enhanced HR rates (Luo et al., 2012). Seedlings of the Arabidopsis *ae7* mutant have shown increased sensitivity to the DNA damage agents, methylmethane sulphonate and cisplatin. The *ae7* mutant cells have also been shown to be blocked at the G2/M transition of the cell cycle and revealed increased expression of DDR genes, including PARP (Poly(ADP-ribose) polymerase), BRCA1, GR1 (Gamma response 1), and TOS2 (Ribonucleotide reductase-like catalytic subunit), involved in DSB repair and genome maintenance (Luo et al., 2012). The defective CIA pathway would seem to cause genotoxic damage, which triggers cell cycle arrest and DDR. Similarly, increased sensitivity to genotoxic agents and up-regulation of DDR genes have been observed in the Arabidopsis *atm3* mutant lacking the ATM3 function (Luo et al., 2012).

Chromatin remodeling is also a key aspect since it is necessary for the access of the DDR protein to the damaged DNA site. Evidence of the redox-mediated modulation of chromatin remodelers has been provided in animal systems. Duquette et al. (2018) reported that lysine demethylase 1 (LSD1/KDM1A), a flavin adenine dinucleotide (FAD)-dependent amine oxidase able to demethylate the lysine 4 residue of histone H3, triggers H_2_O_2_ accumulation as a by-product of its chromatin remodeling activity during the early steps of DDR. This is the first evidence that ROS can be generated *ex novo* in human cells as part of DDR, at a specific damaged site. In addition, the local production of H_2_O_2_ can control the activity of DNA repair enzymes recruited at the lesion. This suggests that the local redox environment might modulate the two major DBS repair pathways, namely HR and NHEJ (Duquette et al., 2018). It is possible that a similar mechanism also takes place in plant cells. The Arabidopsis genome encodes four LSD1 homologs named LSD1-like (LSDL), of which LSDL1 and LSDL2 control histone H3 methylation only around and within the heterochromatin region containing the floral repressors FLC (FLOWERING LOCUS) and FWA, which is crucial for the timing of the developmental transition to flowering (Jiang et al., 2007). Unlike for animals, there is currently no evidence of the role of plant LSD1-like proteins in DDR.

In the complex and variegated scenario of intersecting DDR and redox mechanisms, it is also possible that the same protein fulfills a dual role, acting in a redox context as well as maintaining genome stability. In the PARP-like genes, found in eukaryotes, the PARP catalytic domain is associated with other functional domains (Vainonen et al., 2016). The Arabidopsis protein RCD1 (inactive poly [ADP-ribose] polymerase) contains a WWE domain (Trp-Trp-Glu, involved in protein-protein interactions occurring in ubiquitination and ADP ribosylation) (Aravind, 2001) and an RST (RCD-SRO-TAF4) domain also responsible for protein-protein interactions. Proteins that contain this domain combination, specific to plants, are named SIMILAR TO RCD-ONE (SRO) (Jaspers et al., 2010). According to Liu et al. (2014), overexpression of the *TaSRO* gene in Arabidopsis provides increased tolerance to genotoxic stress induced by UV irradiation and H_2_O_2_ treatments. The authors ascribed genome integrity to the enhanced PARP activity detected in the *TaSRO*-overexpressing cells that positively affected DDR, resulting in higher levels of the ATM ROS sensor. Interestingly, the *TaSRO*-overexpressing cells accumulated more ROS than the control lines, under both non-stressed and stressed conditions, combined with an efficient antioxidant response that ensured redox homeostasis (Liu et al., 2014). Thus, the particular structural features of *TaSRO* enable this protein to play a dual role in the stress response, acting through the modulation of redox parameters and genome maintenance.

Arabidopsis *apx1/cat2* double mutants that constitutively activate DDR at a transcriptional level represent an interesting example of redox-DDR interaction (Vanderauwera et al., 2011). This confers tolerance against various stresses in the double mutants, since the induced DDR is active also in the absence of DNA damage. DDR induction was inhibited under high CO_2_ in the double mutants, suggesting that the ROS production derived from photorespiration caused DDR induction at a constitutive level in the double mutants also under standard conditions. In addition, the WEE1 serine/threonine kinase-dependent cell cycle checkpoint was activated in *apx1/cat2* mutants, which suggests that cell cycle arrest is part of the signaling pathway activated by ROS involving DDR induction (Vanderauwera et al., 2011).

## Role of Redox Balance in Transcriptional Control

### Redox Regulated Transcriptome Re-programming

Redox-based mechanisms play a key role in the regulation of gene expression. Several studies based on omics approaches have demonstrated that ROS induce transcriptional modifications by direct or indirect mechanisms. This experimental evidence has been mainly obtained by manipulating cell ROS levels and/or redox balance in pharmacological or genetic contexts. Effective case-by-case studies were obtained by using mutants defective in enzymatic antioxidant systems (such as catalase, ascorbate peroxidases and ascorbate oxidase – AO; Vanderauwera et al., 2005; Gadjev et al., 2006, 2006; Rasool et al., 2017) as well as treatments with ROS-generating systems, with electron transfer inhibitors in chloroplast and mitochondria or oxidative stress triggering agents (Gadjev et al., 2006; Broda and Van Aken, 2018). The transcriptomic changes appear to be finely tuned depending on ROS types and production site within the cell (Locato et al., 2018). In fact, various environmental backgrounds can promote ROS increases, above all in the apoplast, chloroplasts, mitochondria, and peroxisomes. To give some examples: biotic stresses as well as high light (HL), salt, and drought have been related to apoplastic ROS accumulation by the activation of plasma membrane-located NADPH oxidases belonging to the family of respiratory burst oxidase homolog proteins (RBOH; Ma et al., 2012; Kadota et al., 2015; Kurusu et al., 2015; Li et al., 2015; Evans et al., 2016; He et al., 2017; Karpinska et al., 2018); HL also induces chloroplast ROS production (Foyer and Shigeoka, 2011), whereas photorespiration mainly causes ROS production in the peroxisomes (Foyer et al., 2009), and a number of abiotic stresses increase ROS production in mitochondria (Foyer and Noctor, 2003). Controlled fluxes of redox active molecules (oxidants and antioxidants) between organelles and cytosol, regulate redox mechanisms which, in turn, results in the control of gene expression within the nuclei (Locato et al., 2018). This gene expression reprogramming possibly enable plants to by-pass a stressful situation or a metabolic impairment. Sewelam et al. (2014) demonstrated that hydrogen peroxide (H_2_O_2_) production triggered by the activation of photorespiratory pathway induced a different set of nuclear genes depending on the ROS production site. Their study used Arabidopsis plants overexpressing glycolate oxidase 5 (GO5), producing ROS in the chloroplast under photorespiratory conditions, and a catalase defective line (*cat2-2*), where ROS over-production occurred in the peroxisomes during photorespiration. When ROS were mainly produced in the chloroplasts, the induced genes mostly belonged to the functional categories of the transcription factors (TFs), proteins involved in signaling and metabolic pathways, and in defense or detoxification. Differently, peroxisome-derived ROS mainly induced the expression of genes involved in protein folding and repair (such as chaperones and heat shock proteins – HSPs), along with defense and detoxification processes. Therefore, different ROS responsive genes were identified to be linked to redox impairment occurring in specific intracellular contexts. A meta-analysis (Vandenbroucke et al., 2008) revealed that yeast, plants, and animals share at least four families of H_2_O_2_-responsive genes: a class of HSPs, GTP-binding proteins, Ca^2+^-dependent protein kinases, and ubiquitin-conjugating enzymes. Antioxidant genes shows an H_2_O_2_-dependent up-regulation only in prokaryotes. This probably depends on the fact that in eukaryotes antioxidant genes show a high constitutive expression probably as an evolutionary acquisition. Thus, in eukaryotes, antioxidant systems are mainly controlled at post-translational level (Vandenbroucke et al., 2008). For example, the synthesis of GSH is controlled by post-translational modification based on a thiol switch mechanism. Oxidative conditions (also determined by stressful conditions) activate the enzyme that catalyzes the limiting biosynthetic step in GSH production, γ-Glutamyl cysteine synthetase (γ-ECS), by disulphide bond formation in the γ-ECS homodimer. This also represents a controlled redox loop involving GSH (reviewed by Yi et al., 2010).

Glutathione is a major redox soluble metabolite controlling the cell redox balance under physiological and perturbed situations in both developmental and defense contexts (Noctor et al., 2012; Hernández et al., 2017; Locato et al., 2017). The effect of GSH on the transcriptome has been investigated in various studies (Cheng et al., 2015 and references therein; Hacham et al., 2014). GSH feeding of Arabidopsis seedlings appears to induce the expression of stress-related genes and down-regulates developmental correlated genes (Hacham et al., 2014). High GSH levels have also often been correlated to increased stress tolerance (Cao et al., 2017; Ferrer et al., 2018; Formentin et al., 2018). Conversely, GSH deficiency in Arabidopsis root meristem less 1 mutant (*rml1*) has been shown to affect root growth and architecture through a massive transcriptome re-programming. This result has been confirmed in other GSH deficient mutants (*cad2-1*; *pad2-1*; *rax1-1*) presenting different mutations in the GSH1 gene, encoding the GSH biosynthetic enzyme γ-ECS, and in condition of GSH depletion by treatment with buthionine sulfoximide (BSO), an inhibitor of GSH biosynthesis. In all these contexts, GSH deficiency affected above all the expression of those genes involved in cell cycle progression, especially those involved in G2-M transition. On the other hand, the expression of several genes related to redox signaling were less modified probably because the GSH redox state did not change in the mentioned experimental conditions. On the other hand, heat shock (HS) responsive genes were down-regulated, suggesting that the lack of GSH affected redox signaling leading to the expression of these genes. This suggests that GSH is generally required in the induction of oxidative-stress related genes. The redox state of nucleus and cytosol in Arabidopsis root cells has also been monitored in *rml1* mutants and in wild type under BSO treatment. In both compartments, the GSH depletion triggered an increase in the redox state, suggesting that the link between root development, growth, and cell redox state is strongly dependent on the GSH level controlling transcriptome re-programming (Schnaubelt et al., 2015). Karpinska et al. (2017) demonstrated that the nuclear redox state is also prone to oxidation when different plant tissues and cell types were treated with inhibitors of mitochondrial and chloroplast electron transfer, which enable oxidative impair within the cells. The authors observed transcriptome re-programming as a consequence of nucleus oxidation, leading to the retrograde regulation of the expression of genes, mainly related to organelle functions. The GSH-dependent control of the nuclear redox state thus appears to be crucial in interconnected signaling networks which are involved in the organelle cross-talk determinant for gene expression regulation. It has also been demonstrated that an increase in GSH, obtained by exogenous treatment or genetically, enhances the translational efficiency of Arabidopsis plants. This enhancement can be inferred from the changes observed in the polysomal fraction profile, which is indicative of the number of active translation events. An increase in the GSH level seems to activate the translation of pre-existing mRNAs of cluster genes related to hormone biosynthesis, proline biosynthesis, stress response, including TFs involved in defense response, root growth, cell cycle, metabolism, and sulfur assimilation. These data are in accordance with the protective role of GSH supplementation against a plethora of different stress conditions. It also suggests an overall control of the translatome and transcriptome of GSH in plants, probably also correlated to the control played by this metabolite in development and cell proliferation (Cheng et al., 2015; Locato et al., 2015).

Another non-enzymatic antioxidant molecule which intracellular concentration affecting the cellular redox state is the ascorbate (ASC). The ASC level and redox state have been correlated to cell proliferation (de Pinto et al., 1999; Pellny et al., 2009; de Simone et al., 2017; Kka et al., 2018), plant development (Foyer and Noctor, 2009; Paradiso et al., 2012; Cimini et al., 2015) and defense (Kiddle et al., 2003; Sabetta et al., 2019). In fact, ASC treatment of quiescent center cells re-activated the cell division process (Liso et al., 1988). However, according to the literature, the possible involvement of ASC in the control of transcriptional events has not been characterized as well as it has been for GSH. A recent system biology study (Stevens et al., 2018) investigated the effect of ASC metabolism perturbation on the transcriptomes, metabolomes, and proteomes of tomato fruits. The study took into account the RNAi lines for AO, L-Galactono-1,4-γ-lactone dehydrogenase (GLD) and monodehydroascorbate reductase (MDHAR), which are all enzymes involved in the control of ASC levels and the redox state. Although in this study the analysis was carried out on a particular non-photosynthetic tissue and reported no differences in metabolite and protein levels, it did reveal the role of the ASC pool in controlling those core genes involved in ribosome biogenesis, structure, translation, and protein folding (Stevens et al., 2018). Another study performed a comparative analysis of the leaf transcriptome of Arabidopsis mutants which showed reduced levels of GSH (*rml1*), ASC (*vitc1*, *vitc2*) and ROS detoxification in peroxisomes (catalase 2 defective mutant; *cat2*) and chloroplasts (thylacoydal ascorbate peroxidase defective mutant; *tapx*) (Queval and Foyer, 2012). It revealed that both low GSH and ASC caused significant transcriptome reprogramming, although deficiencies in the two antioxidants seemed to affect different sets of genes. Interestingly, there was a 30% overlap among the sets of genes regulated by low antioxidant levels and impairment of ROS detoxification systems; whereas only 10% of the genes regulated by H_2_O_2_ increases observed in *cat2* and *tapx* mutants overlapped (Queval and Foyer, 2012).

### The Role of Redox Sensitive TF Regulation in DNA Transcriptional Control

Reactive oxygen species can regulate gene expression by modulating the activity of numerous TFs. Several redox-dependent mechanisms controlling TF activity have been described in plants, although this is still an under-investigated field. Redox regulation may include conformational changes in TFs and TF-binding proteins (positive or negative regulators), or an alteration in their intracellular compartmentalization as well as redox-dependent TF proteolysis. Table 1 summarizes the information related to 12 redox-regulated TFs that directly target several genes involved in plant stress responses. A more detailed description of these TFs and their mechanism of action is provided in the sub-chapters below.

**TABLE 1:** List of redox sensitive TFs and their regulatory mechanism.

**TF’s family**	**TF**	**Redox regulatory mechanism**	**References**
ERF/AP2 TFs	RRTF1	Phosphate-dependent nuclear re-localization of WRKY40 that activate RRTF1 gene expression	Jaspers and Kangasjärvi, 2010; Pandey et al., 2010; Heller and Tudzynski, 2011; Jiang et al., 2011; Matsuo et al., 2015
	RAP2.4a	Conformational state: protein homo-dimerization	Shaikhali et al., 2008; Hiltscher et al., 2014
	RAP2.12	Redox control of the interaction with a binding partner and nuclear re-localization	Gibbs et al., 2011; Licausi et al., 2011; Licausi, 2013; Weits et al., 2014; Kosmacz et al., 2015
ZF-TFs	SRG1	Post-translational modification and redox control of the interaction with a co-repressor	Cui et al., 2018
	ZAT12	Gene expression induction and proteolytic degradation depending on ROS intracellular levels	Davletova et al., 2005; Brumbarova et al., 2016; Le et al., 2016; Xu et al., 2017
bZIP-TFs	PAN	Redox-sensitive DNA-binding controlled by disulphide bridge formation and post-translational modification	Li et al., 2009; Gutsche and Zachgo, 2016
	VIP1	Nuclear re-localization dependent on redox-sensitive interaction with a negative regulator	Takeo and Ito, 2017
	TGA1	Redox-dependent conformational change of the co-activator protein NPR1 that allow its nuclear re-localization and interaction with TGA TFs	Tada et al., 2008; Lindermayr et al., 2010; Kneeshaw et al., 2014; Kovacs et al., 2015
NAC TFs	VND7	Post-translational oxidative modification that affect TF’s transactivation activity	Kawabe et al., 2018
HSFs	HSFA8	Redox-dependent conformational change required for nuclear re-localization	Giesguth et al., 2015
	HSFA4A		Pérez-Salamó et al., 2014
	HSFA6B		Yoshida et al., 2010; Huang et al., 2016

#### Redox Sensitive TF Belonging to the ERF/AP2 TF Family

Different proteins belonging to the ERF/AP2 TF family undergo redox regulation. Of these TFs, the Redox Responsive Transcription Factor1 (RRTF1) seems to be involved in redox homeostasis under adverse conditions. The RRTF1 transcript levels were shown to be strongly and rapidly increased in response to singlet oxygen and other ROS as well as biotic- and abiotic-induced redox signals such as aphid infection, HL, and salt stress exposure (Matsui et al., 2008; Jaspers and Kangasjärvi, 2010; Heller and Tudzynski, 2011; Jiang et al., 2011). The regulation of the activity of this TF is still not well understood. An increase in RRTF1 expression was found after *Alternaria brassicacea* infection and/or H_2_O_2_ treatment. In this context, WRKY18/40/60 has been shown to be required for this up-regulation (Matsuo et al., 2015). In particular, a dynamic sub-nuclear re-localization of WRKY40 is induced by abscisic acid (ABA) treatment in a phosphorylation-dependent manner. Once in the nucleus, WRKY40 binds the promoter region of RRTF1 thereby controlling its gene expression (Pandey et al., 2010). RRTF1 binds to GCC-box-like motifs located in the promoter of RRTF1-responsive genes, thereby favoring an increased defense response under constraint conditions (Matsuo et al., 2015).

The Related to Apetala-2 (RAP2) TFs are also one of the main groups of redox regulated proteins belonging to the ERF/AP2 family. The Arabidopsis RAP2.4 TF class consists of eight members characterized by highly conserved DNA-binding domains with overlapping and specific functions. These RAP2.4 proteins constitute a regulative network in which RAP2.4a is the transcriptional activator of chloroplast peroxidase activity. Other RAP2.4 proteins may function as important modulators since an imbalance in the RAP2.4 pattern can, either positively or negatively, affect the expression of target genes by altering the RAP2.4a transcription (Rudnik et al., 2017). The RAP2.4a TF undergoes dimerization under slightly oxidizing conditions and regulates the induction of three chloroplast peroxidases, namely 2-Cys peroxiredoxin A (2CPA), thylakoid and stromal ascorbate peroxidase (tAPX and sAPX), as well as other enzymes involved in redox homeostasis, such as CuZn-superoxide dismutase (SOD; Shaikhali et al., 2008). Under severe oxidative stress, RAP2.4a oligomerizes, thus suppressing its DNA-binding affinity and consequently reducing the expression of target genes (Shaikhali et al., 2008). The interaction of RAP2.4a with RADICAL INDUCED CELL DEATH 1 (RCD1) supports the activation of RAP2.4a transcriptional activity (Hiltscher et al., 2014).

Another member of the ERF/AP2 TF family involved in the regulation of gene expression in a redox dependent manner is RAP2.12. This TF is anchored at the plasma membrane within an Acyl-CoA binding protein 1 and 2 (ACBP1/2) under aerobic conditions (Gibbs et al., 2011). Upon hypoxia, the interaction RAP2.12-ACBP1/2 is suppressed and RAP2.12 is translocated to the nucleus by a mechanism involving an N-terminal cysteine (Cys). Once inside the nucleus, RAP2.12 activates the expression of hypoxia-responsive genes, such as pyruvate decarboxylase 1 (PDC1) and alcohol dehydrogenase 1 (ADH1) (Licausi et al., 2011). After re-oxygenation, RAP2.12 is subjected to a redox-dependent proteolysis via the oxygen-dependent branch of the N-end rule pathway (Licausi et al., 2011; Licausi, 2013; Kosmacz et al., 2015). An oxygen-dependent oxidation of the penultimate Cys residues at the N-terminus of RAP2.12 occurs under normoxia conditions. This reaction, catalyzed by plant Cys oxidases, leads to RAP2.12 destabilization (Weits et al., 2014).

#### Redox Sensitive TF Belonging to the Zinc Finger TF Family

Proteins belonging to the zinc finger TF (ZF-TFs) family can also be redox-regulated. For example, the ZF-TF SNO-regulated gene1 (SRG1), which has been proposed as a nuclear nitric oxide (NO) sensor (Cui et al., 2018). NO is a reactive signaling molecule that modulates the expression of defense-related genes. In response to pathogen attack, a nitrosative burst occurs leading to transient NO accumulation. Following pathogen recognition and NO accumulation, SRG1 is expressed and binds a repeated sequence ACTN_6_ACT or ACTN_4_ACT within promoters of genes coding for immune repressors. This ZF-TF contains an EAR domain required for the recruitment of the co-repressor TOPLESS, thus favoring the transcriptional suppression of target immune repressors (Figure 1). An additional increase in NO levels induces the S-nitrosylation of SRG1, above all at Cys87. The SRG1 S-nitrosylation relieves DNA binding and transcriptional repression, thus enabling the expression of negative regulators of plant immunity (Figure 1). The S-nitrosylation of Cys87, and possibly other Cys residues paired to the ZF motifs, may lead to Zn^2+^ release and to conformational changes responsible for the altered activity of this ZF-TF (Cui et al., 2018).

Another redox regulated ZF-TF is the ZINC FINGER OF ARABIDOPSIS THALIANA 12 (ZAT12) which has been suggested to be involved in the abiotic stress signaling network. Under iron (Fe) deficiency conditions, H_2_O_2_ content showed a marked increase, which leads to the establishment of oxidizing conditions. H_2_O_2_ may function as a signaling molecule that induces the transcription of the FER-LIKE IRON DEFICIENCY-INDUCED TRANSCRIPTION FACTOR (FIT). The increase in the H_2_O_2_ content occurs in a FIT-dependent manner. Under prolonged Fe deficiency conditions, H_2_O_2_ reduces FIT transcription and activates the transcription of its direct binding partner ZAT12 (Le et al., 2016). In Arabidopsis, ZAT12 transcription has been shown to be up-regulated as a consequence of superoxide anion (O_2_^–^) treatment (Xu et al., 2017). ZAT12 acts as negative regulator of FIT: in the nucleus, ZAT12 engages FIT through its C-terminal EAR motif in a protein complex thereby altering the balance between active and inactive FIT pools. ZAT12 is also required for the up-regulation of other stress-related genes such, as APX1 and BHLH039 TFs (Davletova et al., 2005; Le et al., 2016). ZAT12 has also been found to undergo proteasome-dependent degradation in the presence of high H_2_O_2_ levels. The EAR motif seems to be crucial for this proteasome-targeting (Brumbarova et al., 2016; Le et al., 2016).

#### Redox Sensitive TF Belonging to the Basic Leucine Zipper TF Family

The basic leucine zipper TF (bZIP-TFs) is another family including TFs that undergo redox control. A representative example of a redox-sensitive TF belonging to this family is the Arabidopsis TF PERIANTHIA (PAN), which regulates flower organ development and, in particular, the formation of floral organ primordia (Running and Meyerowitz, 1996). PAN was found to bind the AAGAAT motif located in the second intron of the floral homeotic protein AGAMOUS (AG) (Maier et al., 2009). The nuclear interaction of PAN with ROXY1, a plant-specific glutaredoxin (GRX), is crucial for petal development in Arabidopsis (Li et al., 2009). PAN strongly interacts with the AAGAAT motif only under reducing conditions, and redox-sensitive DNA-binding is controlled by the activity of five N-terminal cysteines. Under oxidizing conditions, Cys68 and Cys87, two N-terminal cysteines, can form a disulphide bridge which may alter the conformational structure of this TF, thus changing its ability to bind the DNA (Gutsche and Zachgo, 2016; Figure 1). PAN also undergoes redox-dependent post-translational modifications. It has been demonstrated that Cys340, located in a putative transactivation domain, can be S-glutathionylated, thus modifying PAN activity (Figure 1). The S-glutathionylation of Cys340 does not affect the PAN DNA binding activity, however, it might indicate an additional redox-dependent strategy capable of altering TF activity (Li et al., 2009; Gutsche and Zachgo, 2016).

The VIRE2-interacting protein 1 (VIP1) is a TF belonging to the bZIP-TF family whose redox-sensitive regulatory mechanism depends on a subcellular relocation due to an altered interaction with a negative regulator. Under control conditions, VIP1 has three phosphorylated serine residues in the HXRXXS motif. In a phosphorylated state, VIP1 can interact with 14-3-3 proteins in the cytosol, and this interaction might inhibit VIP1 nuclear import. Mechanical and hypo-osmotic stress exposure caused de-phosphorylation of VIP1, which resulted in a dissociation of 14-3-3 proteins thereby favoring its nuclear location (Takeo and Ito, 2017).

TGACG-sequence-specific protein-binding (TGA) TFs are bZIP-TFs involved in the redox-regulated activation of defense responses triggering plant immunity under pathogen attack. In Arabidopsis, the salicylic acid (SA)-dependent responses, activated upon pathogen infection, are mediated by the redox-regulated nuclear translocation of NON-EXPRESSOR OF PATHOGENESIS-RELATED GENES 1 (NPR1) and by an altered interaction between NPR1 and TGA1 and TGA2 TFs (Tada et al., 2008; Kneeshaw et al., 2014). NPR1 is a co-activator protein whose status is tightly controlled by redox changes occurring after pathogen infection or SA treatment (Mou et al., 2003). This protein is kept in the cytosol in a disulphide-bound oligomeric homocomplex. A reduction in the disulphide bond in NPR1 was found to occur in response to SA-induced changes in cellular redox status. The consequent monomerization unmasked a nuclear location signal, which enables the protein to relocate into the nucleus. Thioredoxins h5 and h3 (TRXh5 and TRXh3) reduce the disulphide-binding oligomers thereby favoring NPR1 monomerization and its nuclear translocation (Kneeshaw et al., 2014). In the nucleus, NPR1 seems to interact with TGA TFs and this triggers the expression of defense genes, such as pathogenesis-related protein 1 (PR1) (Tada et al., 2008). NO controls the translocation of NPR1 into the nucleus (Tada et al., 2008) and the DNA binding activity of its interactor protein TGA1 (Lindermayr et al., 2010). The oligomer-to-monomer reaction involves transient site-specific S-nitrosylation. The NO donor S-nitrosoglutathione (GSNO) thus promotes the nuclear accumulation of NPR1, PR1 expression induction and increased GSH concentration upon *Pseudomonas* infection. GSH accumulation has been shown to be crucial not only for cellular redox homeostasis but also for SA accumulation and activation of the NPR1-dependent defense response (Kovacs et al., 2015).

#### Redox Sensitive TF Belonging to the NAC TF Family

A member of the NAC TF family, named VASCULAR-RELATED NAC DOMAIN 7 (VND7), appears to undergo a reversible oxidative modification (Kawabe et al., 2018). VND7 is involved in xylem vessel cell differentiation (Yamaguchi et al., 2008). Kawabe et al. (2018) found that VND7 is S-nitrosylated at Cys264 and Cys320 located in the C-terminal region near the transactivation domains. The increased S-nitrosylation of VND7 suppresses the transactivation activity of VND7. In this context, a critical role is played by GSNO reductase 1 (GSNOR1) which is thought to be responsible for maintaining cellular S-nitrosothiol homeostasis by regulating the equilibrium between S-nitrosylated proteins and GSNO. The phenotypic traits of the recessive mutant suppressor of the ectopic vessel cell differentiation induced by VND7 (*seiv1*), i.e., an inhibited xylem cell differentiation, have thus been attributed to a loss of function mutation in gsnor1. Consequently, cellular redox state perception by GSNOR1 seems to be important for cell differentiation in Arabidopsis by regulating the post-translational oxidative modification of the TF VND7 (Kawabe et al., 2018).

#### Redox Sensitive TF Belonging to the HSF, WYRKY, and MYB TF Families

Typical redox sensitive TFs may also be recruited in response to specific adverse environmental situations, for example heat shock factors (HSFs) which activate protective genes in plants subjected to high temperatures or other stress conditions. HSFs recognize the heat stress elements (HSEs) located in promoters of heat-induced targets. Plants have numerous classes of HSFs that are encoded by 21 genes in Arabidopsis (Scharf et al., 2012). HSFs remain inactive in the cytosol by interaction with HSPs. This interaction masks the nuclear location signal and the oligomerisation domain. Under stress conditions, HSPs act as molecular chaperones and HSFs oligomerize and are translocated into the nucleus where they modulate the expression of target genes (Hahn et al., 2011; Mittler et al., 2012; Scharf et al., 2012). A redox-dependent translocation of HSFA8 from the cytosol to the nucleus has been described in Arabidopsis plants subjected to H_2_O_2_ treatment (Giesguth et al., 2015). Two Cys residues act as redox sensors in AtHSFA8: Cys24, which is located in the DNA binding domain, and Cys269, which is located in the C-terminal part of the protein. Disulphide bond formation between Cys24 and Cys269 may cause a drastic conformational change and induce AtHSFA8 translocation into the nucleus probably by its release from multi-heteromeric complexes (Figure 1). In single mutants (AtHSFA8-C24S and AtHSFA8-C269S) and in the double mutant (AtHSFA8 C24/269S), HSFA8 nuclear translocation is thus suppressed under oxidative stress (Giesguth et al., 2015). Similarly, Arabidopsis HSFA4A, described as an H_2_O_2_ sensor, has been reported to form homodimers (or homotrimers). This mechanism is thought to be required for the transactivation activity of this TF (Pérez-Salamó et al., 2014). HSFA4A expression is enhanced by numerous adverse conditions known to induce ROS accumulation such as salt, paraquat, heat/cold treatment, drought, hypoxia and several pathogens (Sun et al., 2001; Libault et al., 2007; Peng et al., 2012). HSFA4A, in turn, seems to modulate transcriptional activation of a set of target genes involved in mounting defense responses to abiotic and biotic stress conditions, such as APX, HSP17.6A, ZAT6, ZAT12, CTP1, WRKY30, and CRK13. In Arabidopsis-related species, the formation of redox-sensitive disulphide bonds of Cys residues may be a requirement for HSFA4A homodimerization. In addition, Ser309, located between two activator domains, has been identified as the preferential phosphorylation site catalyzed by MPK3 and MPK6 (Pérez-Salamó et al., 2014).

HSFA6B is another redox-regulated HSF which might play a role in the ABA-dependent pathway under salt and dehydration (Yoshida et al., 2010; Huang et al., 2016). HSFA6B is a protein located in both the cytosol and nucleus under normal growth conditions. After ABA or leptomycin treatment, there is an increase in its nuclear location. In the nucleus, HSFA6B may interact with other HSF proteins such as HSFA1A, HSFA1B, and HSFA2, thereby forming hetero-oligomeric complexes and significantly activating the transcriptional activity of defense genes such as HSP18.1-CI, DREB2A, and APX2 (Huang et al., 2016). HSFA6B seems to have a functional redundancy with the HSFA6A protein during salt and drought stresses. HSFA6A is present at the nucleus and cytosol simultaneously under physiological conditions. However, after salt and drought treatment, HSFA6A has been mainly detected in the nucleus. HSFA6A functions as a transcriptional activator of target genes involved in the enhancement of stress tolerance by its C-terminal moiety. This TF is, in turn, transcriptionally activated by various TFs such as ABF/AREB proteins, MYB96, MYB2, MYC2, and WRKY TFs under salt and drought stress in Arabidopsis (Abe et al., 2003; Seo et al., 2009; Niu et al., 2012; Hwang et al., 2014). In addition, the VOZ1 protein may interact with the DNA-binding domain of HSFA6A under normal growth conditions; however, under high salinity conditions, VOZ1 expression slightly decreases together with its protein content. Thus, freed from interaction with VOZ1, HSFA6A protein can function as a positive regulator of the gene expression involved in tolerance acquisition (Hwang et al., 2014).

A core set of ROS-responsive transcripts has been identified in the systemic acquired acclimation response of Arabidopsis following HL application. Four different TFs, namely GATA8, WRKY48, WRKY53, and MYB30, were found to control HL-dependent transcriptome re-programming. The expression of these TFs peaked 2 min after HL exposure both in local and systemic leaves. They were found to be associated with ROS/Ca^2+^ waves generated under these stress conditions (Zandalinas et al., 2019). MYB30 also regulates oxidative and heat stress responses by modulating cytosolic Ca^2+^ levels in response to H_2_O_2_ variations through annexin expression modulation. During ROS/Ca^2+^ wave propagation, MYB30 binds the promoters of ANN1 and ANN4, and represses their expression thereby regulating cytosolic Ca^2+^ levels (Liao et al., 2017). WRKY48 and WRKY57 are involved in pathogen- and drought- induced defense responses (Xing et al., 2008; Van Eck et al., 2014; Sun and Yu, 2015) and GATA8 acts as a positive regulator of Arabidopsis seed germination (Liu et al., 2005).

The examples discussed above suggest that under biotic and abiotic stress conditions, ROS cause drastic changes in nuclear gene expression by altering the activity of specific TFs that regulate the synthesis of proteins related to plant stress adaptation.

## The Implication of Mirnas in Redox- and DDR-associated Pathways

Gene expression can be modulated also at post-transcriptional level. At this regard, miRNAs, a type of short non-coding RNAs, have been indicated as promising candidates in the precise regulation of genes by targeting messenger RNAs (mRNAs) for cleavage or directing translational inhibition. miRNAs are generally produced from a primary miRNA transcript, the pri-miRNA, through the activity of nuclear RNase DICER-LIKE 1 (DCL1), while mature miRNAs are incorporated into a protein complex named RISC (RNA-induced silencing complex) (Figure 1; Reinhart et al., 2002; Bartel, 2004).

In human cells, recent studies have investigated the interactions between DDR components, redox signaling pathways, and miRNAs (for reviews see Hu and Gatti, 2011; Wan et al., 2014; Bu et al., 2017). An interplay between miRNAs, DDR and redox signaling pathways is possible. Indeed, both DDR and redox signaling can modulate miRNA expression, while miRNAs can directly or indirectly modulate the expression of proteins that are part of DDR and redox signaling. Understanding the roles of miRNAs in DDR and redox signaling along with their implications in complex diseases such as cancer (He et al., 2016; Arjumand et al., 2018), or throughout the aging process (Bu et al., 2016, 2017), are viewed as diagnostic tools or alternative therapeutic treatments (Huang et al., 2013; Badiola et al., 2015).

The situation is quite different in plants, where only very few studies have started to address this complex picture. Yet, miRNAs have been extensively studied in terms of stress responses and exhaustive reviews regarding this aspect are available (Noman et al., 2017; see recent reviews by Djami-Tchatchou et al., 2017; Wang et al., 2017; Islam et al., 2018). To understand the roles of miRNAs within the redox balance-DDR-miRNA triangle, recent literature was consulted to examine the direct or indirect implication of miRNAs in ROS production/scavenging and DDR pathways, based on their predicted/validated target genes.

### miRNAs and ROS

As above described, ROS are by-products of cellular metabolic processes that can act as secondary messengers in specific signaling pathways. In humans, miRNAs targeting central regulators of the ROS signaling pathway have been identified, such as the Nuclear Factor Erythroid-Derived 2-Like 2 (Nrf2), or Tumor Necrosis Factor-Alpha (TNFa), and ROS scavengers, such as SOD or CAT (Wang et al., 2014). Similarly, studies in plants have revealed the presence of miRNAs targeting genes involved in ROS production and scavenging (Table 2). The influence of miRNAs in these processes can be classified as (1) direct, when directly targeting genes coding for proteins with oxidant or antioxidant properties, and (2) indirect, when the targeted genes affect redox signaling pathways downstream. miR529 is an example of an indirect influence. This miRNA targets some of the genes belonging to the SQUAMOSA promoter-binding protein-like proteins (SPLs), a plant specific transcription factor involved in regulating plant growth and development (Rhoades et al., 2002). Recently developed rice lines overexpressing *MIR529a* have been shown to have increased resistance to oxidative stress imposed by applying exogenous H_2_O_2_, because of enhanced levels of SOD and peroxidase (POD, POX) enzymes (Yue et al., 2017). The authors demonstrated that the over-accumulation of miR529a resulted in an enhanced seed germination rate, root tip cell viability, chlorophyll retention, and reduced leaf rolling rate during exposure to H_2_O_2_. Regarding the miR529a targets, out of the five predicted genes (*OsSPL2*, *OsSPL14*, *OsSPL16*, *OsSPL17*, *OsSPL18*) only two, *OsSPL2* and *OsSPL14*, were downregulated in seedlings overexpressing *MIR529a*, therefore suggesting that these two were the direct targets of miR529a. This also induced the upregulation of other stress-related genes such as *OsCPR5* (Constitutive expression of pathogenesis-related genes 5), proline synthase (Os10g0519700), amino acid kinase (LOC_Os05g38150), peroxidase precursor (LOC_Os04g59150), and *OsVPE3* (Vacuolar processing enzyme-3). Based on these findings, the authors proposed a potential complex network of miR529a-SPLs-downstream genes in the ROS signaling pathway in response to oxidative stress (Yue et al., 2017).

**TABLE 2:** List of miRNAs targeting genes with roles in ROS production and scavenging.

**miRNA**	**Species**	**Targeted genes**	**Related stress**	**References**
miR395	*Arabidopsis thaliana* *Brassica napus* *Oryza sativa* *Nicotiana tabacum*	*ATPS, SULTR2;1*	Nutrient deficiency Heavy metal	Matthewman et al., 2012; Zhang L. W. et al., 2013; Jagadeeswaran et al., 2014; Panda and Sunkar, 2015; Yuan et al., 2016
miR396b	*Poncirus trifoliata*	*ACO*	Cold	Zhang et al., 2016
miR397	*Arabidopsis thaliana* *Oryza sativa* *Lotus japonicus*	*LAC*	Nutrient deficiency H_2_O_2_	Li et al., 2011; De Luis et al., 2012; Zhang Y. C. et al., 2013; Wang et al., 2014
miR398	*Arabidopsis thaliana* *Vitis vinifera* *Triticum aestivum* *Phaseolus vulgaris* *Medicago truncatula*	*CDS1, CDS2, Nod19, COX5b*	Heavy metal Drought Salinity	Trindade et al., 2010; Naya et al., 2014; Kayıhan et al., 2016; Leng et al., 2017; Li J. et al., 2017; Li L. et al., 2017
miR408	*Arabidopsis thaliana* *Oryza sativa* *Nicotiana tabacum* *Medicago truncatula*	*PCY, PLC, LAC, UCC, UCL8*	Biotic stress Drought Salinity γ-irradiation	Trindade et al., 2010; Zhang et al., 2017; Pan et al., 2018; Song et al., 2018
miR414	*Panicum virgatum*	*CAT isozyme B, PAO, NADH_UbQ/plastoQ_OxRdtase, HSP, COX*	–	Xie et al., 2010
miR474	*Citrus sinensis* *Zea mays*	*PDH, NAD-*dependent malic enzyme	Boron deficiency Submergence	Zhang et al., 2008; Lu et al., 2014
miR477	*Panicum virgatum* *Triticum aestivum*	*Fd-GOGAT*	Drought	Xie et al., 2010; Akdogan et al., 2016
miR528	*Oryza sativa* *Agrostis stolonifera*	*PCY-*like*, LAC, MCOs, GALTs, AO*	Drought Salinity Heavy metals	Liu et al., 2015; Yuan et al., 2015; Wu et al., 2017
miR531	*Panicum virgatum* *Triticum aestivum*	*HSP 17.9, POD52, POX, CYP P450, ACO1*	Environmental pollutants	Xie et al., 2010; Li J. et al., 2017
miR9773	*Triticum aestivum*	*CYP P450*	Environmental pollutants	Li J. et al., 2017
miR1121	*Triticum aestivum*	*CAT-1, POD6, MT3-*like	Environmental pollutants	Li J. et al., 2017
miR9653b	*Triticum aestivum*	*LOX-*like protein	Environmental pollutants	Li J. et al., 2017
miR1132	*Panicum virgatum*	*CYP87A15*	–	Xie et al., 2010
miR1436	*Panicum virgatum Oryza sativa*	*POD2*	Heat	Xie et al., 2010; Mangrauthia et al., 2017
miR1535	*Panicum virgatum*	*CYP724B3*	–	Xie et al., 2010
miR2102	*Panicum virgatum Oryza sativa*	*CYP P450, SOD, COX VI, POD, ACO1*	Arsenic	Xie et al., 2010; Sharma et al., 2015
PC-5p-213179-14	*Zea mays*	*POD*	Low seed vigor	Gong et al., 2015
PN-2013	*Triticum aestivum*	*MDHAR*	Biotic stress	Feng et al., 2014
novel_miR_120	*Brachypodium distachyon*	*NDH1α* subunit 12	H_2_O_2_	Lv et al., 2016
novel_miR_4	*Brachypodium distachyon*	*CYP P450 734A1*	H_2_O_2_	Lv et al., 2016
novel_miR_234	*Brachypodium distachyon*	*FTR*	H_2_O_2_	Lv et al., 2016
novel_miR_197	*Brachypodium distachyon*	*CYP P450 90D2*	H_2_O_2_	Lv et al., 2016

Table 2 summarizes the information related to 23 miRNAs that directly target several genes with roles in ROS production/scavenging in various plant species. Of these, the most studied in relation to oxidative stresses are miR395 and miR398. The predicted and validated targets of miR395 are the ATP sulfurylase (ATPS) and low-affinity sulfate transporters SULTR2;1 (Matthewman et al., 2012; Jagadeeswaran et al., 2014). ATPS catalyzes the activation of sulfate by transferring sulfate to the adenine monophosphate moiety of ATP to form adenosine 5′-phosphosulfate and pyrophosphate (Patron et al., 2008). SULTR2;1 is responsible for the internal transport of sulfate from roots to shoots (Takahashi et al., 2000). The modulation of miR395 thus seems ideal to address the sulfate assimilation pathway and develop crops with increased efficiency of sulfate uptake (Yuan et al., 2016). A key question is how this is related to redox signaling. When sulfate reaches chloroplasts and mitochondria, it is reduced first to sulphite and then to sulfide, which is essential for the synthesis of cysteine and methionine, two fundamental amino acids for supporting redox reactions in plants. The reduced form of cysteine functions as an electron donor, while its oxidized form acts as an electron acceptor. This different redox state allows to hypothesize a role of redox signaling in inducing nutrient-related or stress-responsive miRNAs. Above all, it refers to the intracellular thiol redox status, which regulates a variety of cellular and molecular events such as the activity of proteins, signal transduction, transcription and several other cellular functions (Panda and Sunkar, 2015). Another well-studied example is miR398, which targets the metal-induced superoxide dismutases, CDS1 and CDS2, in a number of different species (see Table 2; Trindade et al., 2010; Naya et al., 2014; Kayıhan et al., 2016; Leng et al., 2017; Li J. et al., 2017; Li L. et al., 2017). Because of its role in regulating this important ROS scavenger enzyme, miR398 has been found to be involved in plant responses to a multitude of stresses, including drought (Trindade et al., 2010), salinity (Feng et al., 2015), metal-induced toxicity (Xu et al., 2013), and other pollutants such as sulfur dioxide (SO_2_) (Li L. et al., 2017).

Other miRNAs (e.g., miR414, miR531, miR1121, miR1436, miR2102) that target other ROS scavenging enzymes such as CAT, SOD, POD, and POX have been identified and their involvement in the plant stress response has been proven (Xie et al., 2010; Sharma et al., 2015; Li J. et al., 2017; Table 2). A particular example is miR414, which targets a myriad of genes with different functions in plant stress metabolism and antioxidant responses. As shown by Xie et al. (2010) in switchgrass, miR414 was predicted to target 44 different mRNAs, several of which dealing with oxygen/ROS including CAT isozyme B, polyamine oxidase (PAO), cytochrome *c* oxidase (COX), and NADH-ubiquinone oxidoreductase B16.6 subunit (NADH_UbQ/plastoQ_OxRdtase).

### miRNAs and DDR

The ability of DDR to sense DNA damage, transduce signals and promote repair, depends on the coordinated action of a series of factors. Of these, the MRN complex represents the first “line of defense” as it acts as a sensor of damage signaling by recruiting DDR-related proteins, including ATM and other mediators, to the DSB sites (Goldstein and Kastan, 2015).

In human cell research, miRNAs are being investigated for their modulator role in the regulation of DDR (for review see Hu and Gatti, 2011; He et al., 2017). For example, miR-18a and miR-412 have been proved to negatively regulate ATM expression and reduce the capacity of DNA damage repair in tumorigenic cells challenged with irradiation or chemotherapy (Song et al., 2011; Mansour et al., 2013). Other studies have demonstrated that miRNAs are involved in the post-transcriptional regulation of p53 (Hu et al., 2010; Kumar et al., 2011), the master-regulator of DDR that drives the fate of the human cell directing it to DNA repair, cell cycle arrest, apoptosis, or senescence. For instance, miR-25 and miR-30d have been shown to interact with p53, and, as a consequence, its downregulation leads to the suppression of some of its target genes (*p21*, *BAX*, *Puma*) resulting in reduced apoptosis (Kumar et al., 2011). Downstream effectors, such as the DNA repair pathways, are also influenced by miRNAs at least in animals, as shown in several studies investigating human cancer cell lines. Moreover, examples of miRNA involvement in NHEJ (e.g., miR-101) or HR repair mechanism (e.g., miR-107, miR-103, miR-222) have been reported in animal and plant cells (Yan et al., 2010; Huang et al., 2013; Neijenhuis et al., 2013). In the case of the hsa-miR-526b, which targets the Ku80 mRNA, in addition to DSB repair, the plant cell cycle progression is also affected in the G_0_/G_1_ phase (Zhang, 2015).

In plants few studies have addressed the potential role of miRNAs in the regulation of DDR-associated genes. Most of this evidence comes from high-throughput transcriptomic studies dedicated to investigating specific stress responses/adaptations. Table 3 summarizes a collection of miRNAs predicted to target several genes with different roles in the DDR pathway.

**TABLE 3:** List of miRNAs targeting genes with roles in DNA damage response.

**miRNA**	**Species**	**Targeted genes**	**Related stress**	**References**
miR1127a	*Triticum aestivum*	SMARCA3L3	–	Sun et al., 2018
miR2275	*Triticum aestivum* *Prunus persica*	CAF1	Drought	Esmaeili et al., 2017; Sun et al., 2018
miR122c-3p	*Triticum aestivum*	XPB2	–	Sun et al., 2018
miR5179	*Citrus sinensis*	MUTL-homolog 1	Mg-deficiency	Liang et al., 2017
miR5261	*Citrus sinensis*	MRE11	Mg-deficiency	Liang et al., 2017
miR528b	*Hordeum bulbosum*	RFA1C	Salinity	Liu and Sun, 2017
miR403	*Helianthus annuus*	AGO1, AGO2	Salinity	Ebrahimi Khaksefidi et al., 2015; Kumar et al., 2018
miR2102	*Panicum virgatum* *Oryza sativa*	TFIID subunit 10	Arsenic	Xie et al., 2010; Sharma et al., 2015
miR477	*Panicum virgatum* *Triticum aestivum*	RAD23	Drought	Xie et al., 2010; Akdogan et al., 2016
novel-mir_222	*Brachypodium distachyon*	TFIID subunit 12	H_2_O_2_	Lv et al., 2016
novel-mir_120	*Brachypodium distachyon*	TFIID subunit 12	H_2_O_2_	Lv et al., 2016
novel-mir_98	*Brachypodium distachyon*	TFIID subunit 12	H_2_O_2_	Lv et al., 2016
novel-mir_69	*Brachypodium distachyon*	RAD50	H_2_O_2_	Lv et al., 2016
novel-mir_147	*Brachypodium distachyon*	SMUBP-2	H_2_O_2_	Lv et al., 2016
novel-mir_4	*Brachypodium distachyon*	SAGA29	H_2_O_2_	Lv et al., 2016
miR414	*Oryza sativa*	OsABP helicase	Salinity γ-irradiation	Macovei and Tuteja, 2012; Macovei and Tuteja, 2013
miR408	*Oryza sativa*	OsDSHCT helicase	Salinity γ-irradiation	Macovei and Tuteja, 2012; Macovei and Tuteja, 2013
miR164e	*Oryza sativa*	OsDBH helicase	Salinity γ-irradiation	Macovei and Tuteja, 2012; Macovei and Tuteja, 2013

In a study on changes in miRNA expression during magnesium (Mg)-induced starvation in oranges roots, the authors collected different miRNAs affecting several functions, ranging from the antioxidant response, adaptation to low-phosphorus and activation of transport-related genes, to DNA repair (Liang et al., 2017). The study identified the MUTL-homolog 1 (MLH1) and MRE11 as targets of miR5176 and miR5261, respectively. The *MLH1* gene is part of the MMR pathway, one of the DNA repair defense systems responsible for maintaining genome integrity during cell division. Previous studies in yeast have identified four MutL homologs that form functionally distinct heterodimers, of which Mlh1/Pms1 and Mlh1/Mlh2 are involved in the correction of different types of DNA mismatches (Wang et al., 1999). In plants, the MLH1 has been less investigated compared with other MutL/MutS homologs. However, interaction between MLH1 and MLH3 has been shown to be required for the formation of double Holliday junctions and normal levels of meiotic crossovers in Arabidopsis plants (Jackson et al., 2006). Thus, identifying a miRNA capable of suppressing the activity of MLH1 would also help to better clarify the functions of this gene. The particular case of miR5176 showed that its induction under Mg-deprived conditions resulted in the activation rather than the inhibition of MLH1 associated with enhanced MMR activity in response to Mg-deficiency (Liang et al., 2017). This could be due to other post-transcriptional modifications or the activation of alternative regulatory mechanisms. In addition, the *MRE11* gene that encodes DNA repair and meiosis proteins belonging to the MNR complex, was identified as being targeted by miR5261, and induced in Mg-deprived roots. In this case, the down-regulation of miR5261 resulted in enhanced levels of MRE11 and, as a consequence, better detection of DNA damage and repair of DSBs.

In another study aimed at determining miRNAs responsive to H_2_O_2_ during seedling development in *Brachypodium distachyon*, a novel miRNA called novel_mir_69 was identified as targeting the *RAD50* mRNA (Lv et al., 2016). Using next generation high-throughput sequencing, a total of 144 known and 221 new miRNAs were identified as being responsive to H_2_O_2_-induced stress in *B. distachyon*. In addition to *RAD50*, other genes with a role in DNA damage repair were shown to be targeted by several other newly identified miRNAs in this study. For instance, the DNA-binding protein encoded by *SMUBP-2* was predicted to be targeted by novel_mir_147, the novel_mir_4 targeting the SAGA-associated factor 29 homolog, while the transcription initiation factor IID (TFIID) was predicted to be targeted by novel_mir_120. The SMUBP-2 is a transcription regulator which also has a 5′ to 3′ helicase activity. Its RH3 helicase domain and AN1-like zinc finger domain have been shown to bind single-stranded DNA (Lim et al., 2012). The SAGA-associated factor 29 homolog is a chromatin reader component of the transcription regulatory histone acetylation (HAT) complex (Kaldis et al., 2011). On the other hand, TFIID is a key component of the transcription pre-initiation complex (PIC), responsible for recognizing and binding to specific promoter DNA sequences (e.g., TATA elements). Studies on yeast have demonstrated that both TFIID and SAGA can be sequentially recruited at the DNA damage site in a differential manner, based on the type of stress induced (Ghosh and Pugh, 2011). For instance, when the methylmethane sulphonate mutagenic agent was used, the induced genes underwent transcription complex assembly sequentially, first through SAGA and then through a slower TFIID recruitment. However, when heat shock was applied, the induced genes used both the SAGA and TFIID pathways rapidly and in parallel. Similarly, studies in plants have demonstrated that TFIID associates with essential proteins involved in DNA repair and chromatin remodeling, such as MRE11 and TAF1 (TATA-binding protein Associated Factor 1, histone acetyltransferase), in an attempt to maintain genome integrity under genotoxic stress conditions (Waterworth et al., 2015).

The fact that miRNAs were predicted to directly or indirectly interact with chromatin remodeling associated genes further adds to the complicated layers of regulation of this complex phenomenon. In a bioinformatics study on switchgrass, TFIID mRNA was predicted to be targeted by miRNAs (miR2102) (Xie et al., 2010). In the same study, another DNA repair gene, namely *RAD23*, was predicted to be targeted by miR477. The *RAD23* gene, encoding for the UV excision repair protein RAD23 homolog A, is involved in the NER pathway. By interacting with several other components of the DNA repair machinery, it also plays an important role in BER DNA damage recognition (Sturm and Lienhard, 1998). In Arabidopsis, RAD23 have also been demonstrated to have an essential role in the cell cycle, morphology, and fertility of plants through their involvement in ubiquitination pathways (Farmer et al., 2010). Another component of the NER pathway predicted to be targeted by miRNAs is *XPB2* (homolog of *Xeroderma pigmentosum* complementation group B2). In a transcriptome analysis performed during anther development in male sterile wheat lines, *XPB2*, a DNA repair helicase, was shown to be targeted by tae-miR1122c-3p (Sun et al., 2018). The induced expression of *XPB2*, acting as a DNA damage detector, has been suggested to be necessary for DNA damage repair during pollen formation. It is worth noting that this study used a particular wheat line (337S), which is sensitive to both long-day-length/high-temperature and short-day-length/low-temperature, to investigate the miRNA involvement in the regulation of male sterility by looking at the pre-meiotic and meiotic cell formation (Sun et al., 2018). Besides *XPB2*, other DNA repair and chromatin remodeling associated genes have been identified as targets of miRNAs. For instance, tae-miR2275 targeted the *CAF1* (CCR4-associated factor 1), involved in early meiosis, whereas tae-miR1127a targeted the *SMARCA3L3* (a new member of SWI/SNF factor SWI/SNF-related matrix-associated actin-dependent regulator of chromatin subfamily A, member 3-like 3), believed to be involved in the progression of meiosis in male reproductive cells. In yeast, the CCR4-Not (Carbon Catabolite Repressed 4-Negative on TATA-less) complex has been shown to be involved in replication stress and DNA damage repair, as well as maintaining heterochromatin integrity (Mulder et al., 2005; Cotobal et al., 2015). The SWI/SNF chromatin-remodeling complex is, instead, an essential component of chromatin remodeling, and its involvement in DNA damage response is dependent on the CCR4–Not complex (Mulder et al., 2005). By showing interactions between tae-miR2275-*CAF1* and tae-miR1127a-*SMARCA3L3*, this study demonstrated that the diversified roles of SMARCA3L3 and CAF1 in DNA repair and chromatin remodeling helped to maintain chromatin and genome integrity during meiosis (Sun et al., 2018).

Other miRNAs that putatively control different helicase genes have been identified by *in silico* analysis in rice (Umate and Tuteja, 2010). Of these, osa-MIR414, osa-MIR408 and osa-MIR164e have been experimentally validated as targeting the *OsABP* (ATP-Binding Protein), *OsDSHCT* (DOB1/SK12/helY-like DEAD-box Helicase), and *OsDBH* (DEAD-Box Helicase) genes (Macovei and Tuteja, 2012). The expression of miRNAs and their targeted genes correlated negatively in response to salinity stress and gamma-irradiation treatments, which caused DNA, damage (Macovei and Tuteja, 2012, 2013). Given that helicases are enzymes that catalyze the separation of double-stranded nucleic acids in an energy-dependent manner, they are involved in a wide range of processes such as recombination, replication and translation initiation, double-strand break repair, maintenance of telomere length, nucleotide excision repair, and cell division and proliferation (Tuteja, 2003). Hence, by targeting a wide range of helicases, as shown by the literature cited here, miRNAs are responsible for regulating all this array of processes associated with helicase activities.

An interesting aspect of miRNAs is their capacity to regulate their own biogenesis. This happens by targeting ARGONAUTE genes (AGO1 and AGO2), as in the case of miR403 and miR172 (Ebrahimi Khaksefidi et al., 2015). Aside from their involvement in small RNA pathways and epigenetic silencing phenomena (Schraivogel and Meister, 2014), AGOs have also been shown to be associated directly or indirectly with DNA repair (Oliver et al., 2014). The particular case of miR403 and miR172 shows that in addition to targeting AGO, they also interact with DML1 and DML3 (involved in DNA methylation), thus suggesting the multiple role of these miRNAs in small RNA pathways and DNA methylation (Ebrahimi Khaksefidi et al., 2015).

## Conclusion

This review has explored the interconnections between the molecular mechanisms controlling the cell redox balance and gene expression regulation, occurring at transcriptional and post-transcriptional levels, as well as the maintenance of genome integrity (Figure 1). In particular, evidence here reported, underline the influence of the redox signaling in the modulation of molecular pathways activated in response to developmental and environmental stimuli. Interestingly, specific players involved in redox sensing and homeostasis, influence plant metabolism at different levels. During evolution, plants, as all other living organisms, have developed capability for using specific molecular players in a cross-cutting manner both in developmental processes, in defense responses activated by environmental stimuli and in DNA replication and repair. GSH and correlated thiol systems represent a case in point of key actors controlling the redox buffering capability of plant nuclei and they are crucial also for DNA replication and repair, and consequently cell cycle progression, as well as for the regulation of gene expression in different contexts (Diaz-Vivancos et al., 2010; Martins et al., 2018; Ratajczak et al., 2019). Moreover, numerous TFs, regulating the expression of genes involved in plant development, DDR or in the activation of stress-related responses, are described to be redox-regulated. The activity of these TFs is mainly influence d by alterations in the cell redox balance, which lead to conformational changes and their possible subcellular re-location. Recently, evidence of the involvement of a continuously increasing number of miRNAs in several processes is opening new scenarios on the complexity of redox signaling and homeostasis. Although some miRNAs targeting genes with different roles related to defense systems, development and DDR pathways have been predicted or validated in different plant species, this field requires further investigation. Interestingly, some miRNAs have been predicted to target genes belonging to the above-indicated pathways. Examples include miR408 and miR414, which target the helicases involved in DNA repair as well as several genes implicated in the redox system (see Tables 2, 3). Similarly, miR528 is predicted to target RFA1C (replication A 70 KDa DNA-binding subunit C), involved in DNA replication and efficient DNA repair and recombination (Longhese et al., 1994), as well as antioxidant-related genes (e.g., phytochromes, oxidases). Switchgrass miR477 has also been shown to target the Rad23 DNA repair associated factor as well as Ferredoxin-Dependent Glutamine-Oxoglutarate Amidotransferase (Fd-GOGAT), acting as electron donor in glutamate metabolism (Xie et al., 2010). The evidence here reported highlight an interconnectivity between the redox and DDR pathways created by a network of miRNAs. Further studies aimed at clarifying these complex regulatory networks are strongly encouraged.

## Author Contributions

AB, AM, and VL drafted the manuscript. SC, CG, AM, and VL wrote the manuscript. SC and AM created the figures and tables. AB and LD revised and critically improved the manuscript.

## Conflict of Interest Statement

The authors declare that the research was conducted in the absence of any commercial or financial relationships that could be construed as a potential conflict of interest.
